# Supplementary material for: Identification of Freezing-Responsive microRNAs and Their Targets in Chinese Jujube by Small RNA and Degradome Sequencing
Source: Int J Mol Sci. 2026 Sep 4;27(17):7912. doi: 10.3390/ijms27177912 (PMC13566890; doi:10.3390/ijms27177912)
Supplement: Supplementary file 1 [file ijms-27-07912-s001.zip › Supplementary Material S2/novel_miR_51.pdf]

Provisional ID	:	novel_miR_51
Score total	:	0.7
Score for star read(s)	:	-1.3
Score for read counts	:	0
Score for mfe	:	0.4
Score for randfold	:	1.6
Score for cons. seed	:	
Total read count	:	5010
Mature read count	:	2845
Loop read count	:	28
Star read count	:	2137

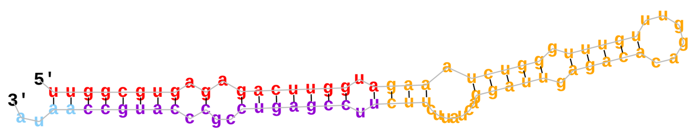

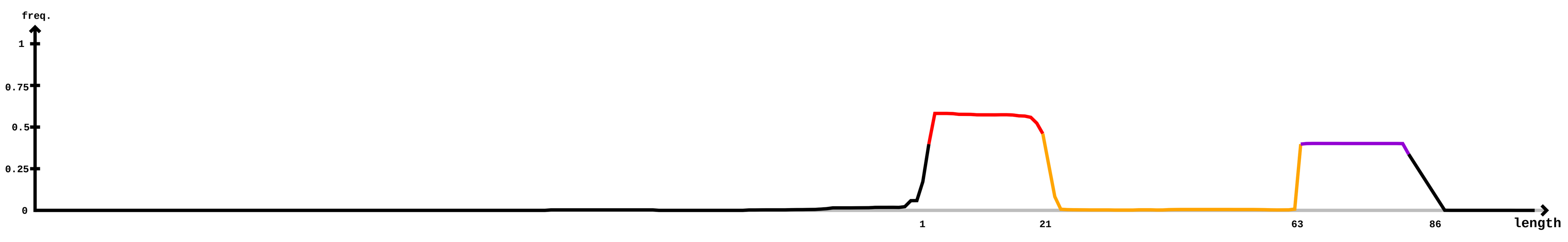

Mature Star

```
5'- agcgucacgugauaacgcguccuuugcuuuuuauuuuuuauuugaaauucaaaaaaauaauaauaaagauauauuuauauaauuguggaugugaaagaagaacuacuaccacuacagagagauuuuuuuaugaggcugagaaaauuguuggcgugagagacuugguagaaaucuggguuuguuuggacacagaguuaggacuauucuucuuccgaguccgcccaugccaauaauuuccuccgucuuuuuc -3'  obs
    agcgucacgugauaacgcguccuuugcuuuuuauuuuuuauuugaaauucaaaaaaauaauaauaaagauauauuuauauaauuguggaugugaaagaagaacuacuaccacuacagagagauuuuuuuaugaggcugagaaaauuguuggcgugagagacuugguagaaaucuggguuuguuuggacacagaguuaggacuauucuucuuccgaguccgcccaugccaauaauuuccuccgucuuuuuc       exp
    .((((.........))))((((((((((((((((.(((.(((((...))))).))).))))).)))).((((((((((....))))))))))..................))))).)))........(((((.(((.((((((((((((((.(.(((((((.((((.(((((.(((((.....))))).)))))......)))).)))))))..).))))))))))))))).))).)))))....
```

read	reads	mm	sample
guggaugugaaagaagaa	6	0	JT-2
guggaugugaaagaagaa	9	0	JT-3
agagauuuuuuuaugaggcugagaa	5	0	JT-2
agagauuuuuuuaugaggcugagaa	8	0	JT-3
agauuuuuuuaugaggcugagaaa	2	0	LT-3
gauuuuuuuaugaggcugagaa	1	0	LT-1
uuuuuaugaggcugagaaaau	1	0	JCK-1
uuuuuaugaggcugagaaaau	1	0	JCK-2
uuuuuaugaggcugagaaaauug	3	0	JCK-2
uuuuaugaggcugagaaaauuguuggcgug	2	0	LT-1
uuuaugaggcugagaaaauuguuggcgug	1	0	JT-1
uuaugaggcugagaaaauug	1	0	LT-1
uuaugaggcugagaaaauuguuggcgu	1	0	LT-1
uuaugaggcugagaaaauuguuggcgu	1	0	LT-2
uaugaggcugagaaaauuguuggcgug	2	0	LT-1
augaggcugagaaaauuguuggc	1	0	LT-1
augaggcugagaaaauuguuggU	1	1	LT-2
augaggcugagaaaauuguuggc	2	0	LT-3
augaggcugagaaaauuguuggcg	3	0	JT-3
augaggcugagaaaauuguuggcg	5	0	JT-2
ugaggcugagaaaauuguugg	3	0	LCK-3
ugaggcugagaaaauuguugg	6	0	LT-2
ugaggcugagaaaauuAuugg	1	1	JCK-3
ugaggcugagaaaauuguuggc	1	0	JCK-3
ugaggcugagaaaauuguuggc	1	0	JCK-2
ugaggcugagaaaauuguuggcgug	1	0	JCK-1
ugaggcugagaaaauuguuggcgugaga	1	0	LT-1
gaggcugagaaaauuguuggc	17	0	LCK-3
gaggcugagaaaauuguuggc	1	0	LT-2
gaggcugagaaaauuguuggcg	2	0	LT-3
gaggcugagaaaauuAuuggcgug	2	1	JCK-3
gaggcugagaaaauuAuuggcgug	1	1	JCK-2
gaggcugagaaaauuguuggcgug	1	0	JCK-1

Mature | Star

agcgucacgugauaacgcguccuuugcuuuuuauuuuuuauuugaaauucaaaaaaauaauaauaaagauauauuuauauaauuguggaugugaaagaagaacuacuaccacuacagagagauuuuuuuaugaggcugagaaaauug**uuggcgugagagacuuggua**gaaaucuggguuuguuuggacacagaguuaggacuauucuuc**uuccgaguccgcccaugcc**aauaauuuccuccgucuuuuuc

(Mature = uuggcgugagagacuuggua, shown in red; loop = gaaaucuggguuuguuuggacacagaguuaggacuauucuuc, shown in orange; Star = uuccgaguccgcccaugcc, shown in purple)

gaggcugagaaaauuguuggcgug	2	0	LT-3
cugagaaaauuguuggcgug	1	0	LT-3
cugagaaaauuguuggcguga	1	0	LT-3
ugagaaaauuguuggcgugagagacu	1	0	LT-3
gagaaaauugAuggcgugagaga	1	1	LT-3
agaaaauuguuggcgugagag	3	0	LT-3
agaaaauuguuggcgugagag	2	0	LT-1
agaaaauuguuggcgugagagacu	6	0	LCK-3
gaaaauuAuuggcgugagagacuuggua	1	1	JCK-1
aaauuAuuggcgugagagacuugg	1	1	JCK-1
aauuguuggcgugagagac	1	0	LT-1
aauuguuggcgugagagac	6	0	LCK-3
aCuuguuggcgugagagacu	1	1	LT-3
aauuguuggcgugagagacuugg	2	0	JCK-3
aauuguuggcgugagagacuugg	1	0	JCK-2
auuguuggcgugagagac	2	0	LT-2
auuAuuggcgugagagacu	1	1	JCK-2
auuguuggcgugagagacuug	1	0	LT-3
auuguuggcgugagagacuug	2	0	LT-1
auuAuuggcgugagagacuuggua	1	1	JCK-1
auuAuuggcgugagagacuuggua	2	1	JCK-2
auuguuggcgugagagacuuggua	3	0	LT-1
auuguuggcgugagagacuuggua	4	0	LT-3
auuAuuggcgugagagacuuggua	2	1	JCK-3
auuguuggcgugagagacuuggua	4	0	LCK-3
auuguuggcgugagagacuuggua	4	0	LT-2
auuguuggcgugagagacuuggua	1	0	JCK-1
uuguuggcgugagagacu	2	0	LT-1
uuguuggcAugagagacu	1	1	LCK-3
uuAuuggcgugagagacu	3	1	JCK-2
uuguuggcgugagagacu	5	0	LCK-3
uuAuuggcgugagagacu	1	1	JCK-3
uuguuggcgugagagacu	2	0	JCK-2
uuguuggcgugagagacu	3	0	LT-3
uuguuggcgugagagacuu	1	0	JCK-1
uuAuuggcgugagagacuu	1	1	JCK-2
uuguuggcgugagagacuu	2	0	LT-1
uuAuuggcgugagagacuu	3	1	JCK-3
uuguuggcgugagagacuug	2	0	LT-2
uuAuuggcgugagagacuug	1	1	JCK-2
uuguuggcgugagagacuug	8	0	LCK-3
uuAuuggcgugagagacuugg	41	1	JCK-2
uuAuuggcgugagagacuugg	29	1	JCK-3
uuCuuggcgugagagacuugg	1	1	JCK-2
uuguuggcgugagagacuugg	10	0	LT-2
uuAuuggcgugagagacuugg	11	1	JT-1
uuguuggcgugagagacuugg	6	0	LT-3
uuAuuggcgugagagacuugg	32	1	JCK-1
uuguuggcgugagagacuugg	5	0	LT-1
uuguuggcgugagagacuugg	1	0	JCK-3
uuguuggcgugagagacuugg	7	0	LCK-3
uuguuggcgugagagacuuggu	1	0	LT-1
uuguuggcgugagagacuuggu	1	0	JCK-2
uuguuggcgugagagacuuggu	1	0	LT-2
uuguuggcgugagagacuuggu	1	0	JCK-1
uuAuuggcgugagagacuuggu	1	1	JCK-1
uuguuggcgugagagacuuggu	1	0	JCK-3
uuguuggcgugagagacuuggua	2	0	LT-3
uuAuuggcgugagagacuuggua	4	1	JCK-3
uuguuggcgugagagacuuggua	1	0	LT-1
uuAuuggcgugagagacuuggua	1	1	JCK-1
uuAuuggcgugagagacuuggua	3	1	JCK-2
uguuggcgugagagacuu	1	0	LT-1
uguuggcgugagagacuugg	1	0	LT-1
uguuggcgugagagacuuggua	2	0	LT-3
Auggcgugagagacuug	6	1	JCK-1
Auggcgugagagacuug	12	1	JCK-2
Auggcgugagagacuug	2	1	JT-1
Auggcgugagagacuug	7	1	JCK-3
Auggcgugagagacuugg	4	1	JCK-2

Mature   Star

agcgucacgugauaacgcguccuuugcuuuuuauuuuuuauuugaaauucaaaaaaauaauaauaaagauauauuuauauaauuguggaugugaaagaagaacuacuaccacuacagagagauuuuuuuaugaggcugagaaaauuguuggcgugagagacuugguagaaaucuggguuuguuuggacacagaguuaggacuauucuucuuccgaguccgcccaugccaauaauuuccuccgucuuuuc

Auuggcgugagagacuugg	3	1	JCK-1
Auuggcgugagagacuugg	7	1	JT-2
Auuggcgugagagacuugg	3	1	JCK-3
Auuggcgugagagacuugg	5	1	JT-3
Auuggcgugagagacuuggu	3	1	JT-3
Auuggcgugagagacuuggu	3	1	JT-2
Auuggcgugagagacuuggu	14	1	JCK-1
Auuggcgugagagacuuggu	55	1	JCK-2
Cuuggcgugagagacuuggu	1	1	JCK-2
Auuggcgugagagacuuggu	24	1	JCK-3
Cuuggcgugagagacuuggua	2	1	JCK-2
Auuggcgugagagacuuggua	81	1	JCK-1
Auuggcgugagagacuuggua	144	1	JCK-3
guuggcgugagagacuuggua	2	0	LT-1
Auuggcgugagagacuuggua	171	1	JCK-2
Cuuggcgugagagacuuggua	2	1	JCK-3
Auuggcgugagagacuuggua	9	1	JT-1
Auuggcgugagagacuugguag	20	1	JCK-1
Auuggcgugagagacuugguag	18	1	JCK-3
Auuggcgugagagacuugguag	17	1	JCK-2
guuggcgugagagacuugguagaa	1	0	LT-3
uuggcgugagagacuugg	2	0	LCK-3
uuggcgugagagacuugg	4	0	LT-2
uuggcgugagagacuugg	3	0	LT-3
uuggcgugagagacuugg	2	0	LT-1
uuggcgugagagacuugg	6	0	JCK-2
uuggcgugagagacuugg	4	0	JCK-3
uuggcgugagagacuuggu	23	0	JCK-3
uuggcgugagagacuuggu	3	0	LCK-3
uuggcgugagagacuuggu	11	0	JT-1
uuggcgugagagacuuggu	20	0	LCK-1
uNggcgugagagacuuggu	1	1	JCK-2
uuggcgugagagacuuggu	41	0	JCK-2
uuggcgugagagacuuggu	15	0	LT-1
uuggcgugagagacuugUu	1	1	JCK-3
uuggcgugagagacuuggu	1	0	JT-3
uuggcgugagagacuuggu	17	0	LT-2
uuggcgugagagacuuggu	7	0	LT-3
uuggcgugagagacuuggu	2	0	JT-2
uuggcgugagagacuuggu	24	0	LCK-2
uuggcgugagagacuuggu	18	0	JCK-1
uuggcgugagagaAuuggu	1	1	JT-2
uuggcgugagCgacuuggua	1	1	JCK-2
uuggcgugagagacuuggua	7	0	JT-2
uuggcgugagagacuuggua	13	0	LT-1
uuggcgugagagacuugCua	1	1	JCK-1
uuggcgugagagacuuggua	208	0	JCK-2
uuggcgugagagacuuAgua	1	1	JCK-2
uuggcgugagagaAuuggua	2	1	JCK-1
uuggcgugagagaAuuggua	1	1	JCK-3
uuggcgugagagacuugguU	4	1	JCK-2
uuggcgugagagacuuggua	6	0	JT-3
uuggcgugagagacuugguC	1	1	LT-1
uuggcgugagagacuuggua	41	0	LCK-3
uuggcgugagagacuugguC	2	1	LT-3
uuggcgugagagacuugguU	3	1	JCK-1
uuggcgugagagacuuggua	32	0	JT-1
uuggcgugagagacCuggua	1	1	JCK-3
uuggcgugagagacuuggua	183	0	JCK-3
uuggcgugagCgacuuggua	2	1	JT-1
uuggcgugagagacuugguU	10	1	JCK-3
uuggcgugagagacuugguU	2	1	LT-3
uuggcgugagagacuuggua	21	0	LT-2
uuggcgugagagacuuggua	154	0	JCK-1
uuggcgugagagacuugguC	1	1	JCK-2
uuggcgugagagacuugguU	4	1	LCK-3
uuggcgugagaUacuuggua	1	1	LT-2
uuggcgugagaAacuuggua	1	1	JCK-2
uuggcgugagagacuuggua	46	0	LT-3
uuggcgugCgagacuuggua	1	1	JT-1

Mature Star

agcgucacgugauaacgcguccuuugcuuuuuauuuuuuauuugaaauucaaaaaaauaauaauaaagauauauuuauauaauuguggaugugaaagaagaacuacuaccacuacagagagauuuuuuuaugaggcugagaaaauuguuggcgugagagacuugguagaaaucuggguuuguuuggacacagaguuaggacuauucuucuuccgaguccgcccaugccaauaauuuccuccgucuuuuuc

uuggcgugagagacuugguag	7	0	LT-3
uuggcgugagagacuugguag	40	0	JT-1
uuggcgugagagacuugguag	31	0	JCK-1
uuggcgugagagacuugguag	27	0	JCK-3
uuggcgugagagacuugguaA	1	1	JCK-2
uuggcgugagagacuugguag	8	0	LT-1
uuggcgugagagacuugguaA	2	1	JCK-1
uuggcgugagagacuugguaA	3	1	JCK-3
uuggcgugagaNacuugguag	1	1	JCK-1
uuggcgugagagacuugguag	13	0	LT-2
uuggcgugagagacuugguag	2	0	LCK-3
uuggcgugagCgacuugguag	1	1	JCK-3
uuggcgugagagacuugguag	62	0	JCK-2
uuggcgugagagacuugguagU	3	1	LT-1
uuggcgugagagacuugguagU	12	1	LT-3
uuggcgugagagacuugguagC	1	1	JCK-1
uuggcgugagagacuugguagU	14	1	JCK-3
uuggcgugagagacuugguagC	1	1	JCK-2
uuggcgugagagacuugguagC	1	1	JT-2
uuggcgugagagacuugguagC	5	1	JT-3
uuggcgugagagacuugguagU	18	1	JCK-1
uuggcgugagagacuugguagU	12	1	JCK-2
uggcgugagagacuuggu	10	0	LCK-3
uggcgugagagacuuggA	2	1	JCK-1
uggcgugagagGcuuggu	1	1	LT-3
uggcgugagagacuugUu	1	1	JCK-2
uggcgugagagacuuggu	8	0	JCK-1
uggcgugagagacuuggu	6	0	LT-2
uggcgugagagacuuggu	6	0	LT-3
uggcgugagagacuuggu	7	0	JCK-3
uggcgugagagacuuggu	6	0	JCK-2
uggcgugagagacuuggu	2	0	LT-1
uggcgugagagacuugguU	2	1	JCK-1
uggcgugagagacuuggua	5	0	JT-3
uggcgugagagacuuggua	41	0	JCK-1
uggcgugagagacuuggua	30	0	LT-1
uggcgugagagacuuggua	41	0	LT-2
uggcgugagagacuugguU	1	1	LT-3
uggcgugagagacuuggua	9	0	JT-2
uggcgugagagacuugguC	1	1	JCK-2
uggcgugagagacuuggua	42	0	LCK-3
uggcgugagagacuugguC	4	1	LCK-3
uggcgugagagacuugguC	2	1	JCK-1
uggcgugagagacuuggua	12	0	JT-1
uggcgugagagacuuggua	51	0	LT-3
uggcgugagagacuugguU	7	1	LCK-3
uggcgugagagacuuggua	47	0	JCK-3
uggcgugagagacuugguU	6	1	JCK-2
uggcgugagagacuuggua	50	0	JCK-2
uggcgugagagaAuuggua	1	1	LT-2
uggcgugagagacuugguag	38	0	JCK-2
uggcgugCgagacuugguag	1	1	JCK-3
uggcgugagagacuugguag	29	0	JCK-3
uggcAugagagacuugguag	1	1	JCK-1
uggcgugagagacuugguag	8	0	LCK-3
uggcgugagCgacuugguag	1	1	LT-3
uggcgugagagacuugguag	26	0	LT-3
uggcgugagagacuugguaA	3	1	LT-2
uggcgugagagacuugguag	13	0	LT-1
uggcgugagagacuugguag	20	0	LT-2
uggcgugagagacuugguaU	1	1	LT-1
uggcgugagagacuugguag	23	0	JT-3
uggcgugagagacuugguag	18	0	JT-2
uggcgugagagacuugguag	35	0	JCK-1
uggcgugagagaAuugguag	1	1	LT-2
uggcgugagagacuugguag	25	0	JT-1
uggcgugagagacuugguaga	14	0	JT-3
uggcgugagagacuugguaga	54	0	LT-2
uggcgugagagacuugguaga	80	0	LT-3
uggcgugCgagacuugguaga	1	1	LT-1

Mature Star

agcgucacgugauaacgcguccuuugcuuuuuauuuuuuauuugaaauucaaaaaaauaauaauaaagauauauuuauauaauuguggaugugaaagaagaacuacuaccacuacagagagauuuuuuuaugaggcugagaaaauuguuggcgugagagacuugguagaaaucuggguuuguuuggacacagaguuaggacuauucuucuuccgaguccgcccaugccaauaauuuccuccgucuuuuc

uggcgugagagacuugguaga	27	0	JCK-2
uggcgugagagacuugguaga	6	0	JT-2
uggcgugagagacuugguaga	29	0	JT-1
uggcgugagagacuugguaga	25	0	JCK-3
uggcgugagagCcuugguaga	1	1	JCK-3
uggcgugagagacuugguagU	1	1	LT-3
uggcgugagagacuugguaga	22	0	JCK-1
uggcgugagagacuugguaga	36	0	LCK-3
uggcgugagagacuugguaga	42	0	LT-1
uggcgugagagacuugguagU	1	1	JCK-2
uggcgugagagacuugguagC	1	1	LT-3
uggcgugagagacuugguagC	1	1	JCK-3
uggcgugagagacuugguagaa	1	0	JCK-2
uggcgugagagacuugguagaa	2	0	JCK-1
uggcgugagagacuugguagaU	4	1	LT-2
uggcgugagagacuugguagaa	1	0	LT-1
uggcgugagagacuugguagaU	4	1	LT-3
cgugagagacuugguaga	1	0	JCK-3
cgugagagacuugguagaaau	1	0	LT-2
cgugagagacuugguagaaauc	1	0	LT-3
cgugagagacuugguagaaaucu	1	0	LT-1
gugagagacuugguagaaauc	2	0	LT-2
ugagagacuugguagaaa	3	0	LT-3
ugagagacuugguagaaa	1	0	LT-1
ugagagacuugguagaaau	1	0	LT-3
ugagagacuugguagaaaucu	1	0	JCK-2
ugagagacuugguagaaaucuggg	2	0	JCK-2
ugagagacuugguagaaaucugggu	1	0	LT-1
acuugguagaaaucugggu	3	0	LT-3
acuugguagaaaucuggguuuguuug	3	0	JT-3
acuugguagaaaucuggguuuguuug	3	0	JT-2
cuugguagaaaucuggguuug	1	0	JCK-3
uggguuuguuuggacacagaguuag	1	0	LT-3
uuuguuuggacacagaguuaggacuau	1	0	JCK-3
uuuguuuggacacagaguuaggacuau	1	0	JCK-2
uuuggacacagaUuuaggacuauu	1	1	LT-3
uuggacacagaUuuaggacu	1	1	LT-1
uuggacacagaguuaggacu	1	0	JCK-3
uuggacacagaUuuaggacua	2	1	LT-2
uuggacacagaUuuaggacua	1	1	LT-1
uuggacacagaguuaggacuau	3	0	LCK-3
acacagaUuuaggacuauucuu	1	1	LT-3
cacagaUuuaggacuauu	2	1	LT-3
cacagaUuuaggacuauucu	2	1	LT-1
cacagaUuuaggacuauucuu	1	1	LT-3
cacagaUuuaggacuauucuu	1	1	JCK-1
cacagaUuuaggacuauucuu	2	1	JCK-3
cacagaUuuaggacuauucuuc	1	1	JCK-3
cacagaguuaggacuauucuucuucc	1	0	LT-3
acagaUuuaggacuauucuucu	1	1	JCK-3
acagaUuuaggacuauucuucu	2	1	JCK-1
cagaUuuaggacuauucuucu	1	1	JCK-3
cagaguuaggacuauucuucuuccgag	1	0	LT-1
uucuuccgaguccgcccaugcc	1	0	LT-3
ucuuccgaguccgcccaug	3	0	LCK-3
ucuuccgaguccgcccaug	1	0	JCK-2
cuuccgaguccgcccaugc	2	0	LT-2
cuuccgaguccgcccaugc	1	0	LT-1
cuuccgaguccgcccaugc	1	0	LT-3
cuuccgaguccgcccaugc	3	0	LCK-3
cuuccgaguccgcccaugU	2	1	LT-1
cuuccgaguccgcccaugcc	1	0	LT-3
cuuccgaguccgcccaugcc	3	0	LT-2
cuuccgaguccgcccaugcc	5	0	LCK-3
cuuccgaguccgcccaugcc	2	0	LT-1
cuuccgaguccgcccaugcca	1	0	LCK-3
cuuccgaguccgcccaugccaaua	3	0	JT-3
cuuccgaguccgcccaugccaaua	5	0	JT-2
uuccgaguccgcccaugU	1	1	LT-1
uuccgaguccgcccaugc	3	0	JCK-3

Mature Star

```
agcgucacgugauaacgcguccuuugcuuuuuauuuuuuauuugaaauucaaaaaaauaauaauaaagauauauuuauauaauuguggaugugaaagaagaacuacuaccacuacagagagauuuuuuuaugaggcugagaaaauuguuggcgugagagacuugguagaaaucuggguuuguuuggacacagaguuaggacuauucuucuuccgaguccgcccaugccaauaauuuccuccgucuuuuuc
.................................................................................................................................................................................................................uuccgaguccgcccaugc.......................   97    0   LCK-3
.................................................................................................................................................................................................................uuccgaguccgcccaugc.......................   2     0   JT-2
.................................................................................................................................................................................................................uuccgaguccgcccaugc.......................   8     0   JCK-1
.................................................................................................................................................................................................................uuccgaguccgcccaugc.......................   11    0   LCK-1
.................................................................................................................................................................................................................uuccgaguccgcccaugc.......................   88    0   LT-3
.................................................................................................................................................................................................................uuccgaguccgcccaugc.......................   44    0   LT-2
.................................................................................................................................................................................................................uuccgaguccgcccaugc.......................   5     0   JT-3
.................................................................................................................................................................................................................uuccgaguccgcccaugc.......................   51    0   LT-1
.................................................................................................................................................................................................................uuccgaguccgcccaugc.......................   5     0   LCK-2
.................................................................................................................................................................................................................uuccgaguccgcccaugc.......................   12    0   JT-1
.................................................................................................................................................................................................................uuccgaguccgcccaugc.......................   10    0   JCK-2
.................................................................................................................................................................................................................uuccgaguccgcccaugcc......................   9     0   JT-1
.................................................................................................................................................................................................................uuccgaguccgcccaugcc......................   14    0   JT-3
.................................................................................................................................................................................................................uuccgaguccgcccaugcc......................   10    0   JT-2
.................................................................................................................................................................................................................uuccgaguccgcccaugcc......................   108   0   LT-2
.................................................................................................................................................................................................................uuccgaguccgcccaugcc......................   119   0   LT-1
.................................................................................................................................................................................................................uNccgaguccgcccaugcc......................   1     1   JCK-1
.................................................................................................................................................................................................................uuccgaguccgcccaugcU......................   16    1   LT-2
.................................................................................................................................................................................................................uuccgaguccgcccaugcc......................   186   0   LT-3
.................................................................................................................................................................................................................uuccgaguccgcccaugcc......................   36    0   JCK-3
.................................................................................................................................................................................................................uuccgaguccgcccaugcA......................   1     1   LT-2
.................................................................................................................................................................................................................uuccgaguccgcccaugcU......................   1     1   JCK-2
.................................................................................................................................................................................................................uuccgaguccgcccaugcc......................   160   0   LCK-3
.................................................................................................................................................................................................................uuccgaguccgcccaugcU......................   6     1   LT-3
.................................................................................................................................................................................................................uuccgCguccgcccaugcc......................   1     1   LCK-3
.................................................................................................................................................................................................................uuccgaguccgcccaugcU......................   8     1   LT-1
.................................................................................................................................................................................................................uuccgaguccgcccaugcU......................   2     1   JCK-1
.................................................................................................................................................................................................................uuccgaguccgcccaugcU......................   10    1   LCK-3
.................................................................................................................................................................................................................uuccgaguccgcccaugcc......................   41    0   JCK-2
.................................................................................................................................................................................................................uuccgaguccgcccaugcc......................   29    0   JCK-1
.................................................................................................................................................................................................................uuccgaguccgcccaugcca.....................   1     0   JT-2
.................................................................................................................................................................................................................uuccgaguccgcccaugccC.....................   1     1   LT-2
.................................................................................................................................................................................................................uuccgaguccgcccaugcca.....................   28    0   JCK-2
.................................................................................................................................................................................................................uuccgaguccgcccaugcca.....................   37    0   JT-1
.................................................................................................................................................................................................................uuccgaguccgcccaugcca.....................   33    0   LT-2
.................................................................................................................................................................................................................uuccgCguccgcccaugcca.....................   1     1   JT-1
.................................................................................................................................................................................................................uuccgaguccgcccaugcca.....................   7     0   JT-3
.................................................................................................................................................................................................................uuccgaguccgcccaugccU.....................   1     1   LT-3
.................................................................................................................................................................................................................uuccgaguccgcccCugcca.....................   1     1   LT-2
.................................................................................................................................................................................................................uuccgaguccgcccaugcUa.....................   1     1   LT-1
.................................................................................................................................................................................................................uuccgaguccgcccaugccU.....................   1     1   LT-2
.................................................................................................................................................................................................................uuccgaguccgcccaugcca.....................   16    0   JCK-1
.................................................................................................................................................................................................................uuccgaguccgcccaugcca.....................   19    0   JCK-3
.................................................................................................................................................................................................................uuccgaguccgcccaugcca.....................   59    0   LCK-3
.................................................................................................................................................................................................................uuccgaguccgcccaugcca.....................   40    0   LT-1
.................................................................................................................................................................................................................uuccgaguccgcccaugcca.....................   65    0   LT-3
.................................................................................................................................................................................................................uuccgaguccgcccaugccC.....................   1     1   JCK-2
.................................................................................................................................................................................................................uuccgaguccgcccaugccaa....................   16    0   LCK-1
.................................................................................................................................................................................................................uuccgaguccgcccaugccaa....................   1     0   JCK-3
.................................................................................................................................................................................................................uuccgaguccgcccaugccaa....................   12    0   LT-3
.................................................................................................................................................................................................................uuccgaguccgcccaugccaa....................   11    0   LT-1
.................................................................................................................................................................................................................uuccgaguccgcccaugccaa....................   24    0   LCK-3
.................................................................................................................................................................................................................uuccgaguccgcccaugccaa....................   3     0   JCK-2
.................................................................................................................................................................................................................uuccgaguccgcccaugccaa....................   13    0   LT-2
.................................................................................................................................................................................................................uuccgaguccgcccaugccaa....................   2     0   JCK-1
.................................................................................................................................................................................................................uuccgaguccgcccaugccaU....................   1     1   LT-1
.................................................................................................................................................................................................................uuccgaguccgcccaugccaa....................   19    0   LCK-2
.................................................................................................................................................................................................................uuccgaguccgcccaugccaau...................   115   0   JCK-2
.................................................................................................................................................................................................................uuccgCguccgcccaugccaau...................   2     1   JCK-2
.................................................................................................................................................................................................................uuccgaguccgcccaugccaau...................   26    0   LCK-1
.................................................................................................................................................................................................................uuccgaguccgcccaugccaau...................   62    0   LT-3
.................................................................................................................................................................................................................uuccgaguccgcccaugccaau...................   91    0   JCK-3
.................................................................................................................................................................................................................uuccgaguccgcccaugccaau...................   48    0   LT-1
.................................................................................................................................................................................................................uucUgaguccgcccaugccaau...................   1     1   JCK-3
.................................................................................................................................................................................................................uuccgaguccgcccaugccUau...................   2     1   JCK-2
.................................................................................................................................................................................................................uuccgaguccgcccaugccaau...................   19    0   LT-2
.................................................................................................................................................................................................................uuccgCguccgcccaugccaau...................   1     1   JCK-3
.................................................................................................................................................................................................................uuccgaguccgcccaugccaau...................   14    0   LCK-2
.................................................................................................................................................................................................................uuccgaguccgcccaugccaau...................   76    0   JCK-1
.................................................................................................................................................................................................................uuccgaguccgcccaugccUau...................   1     1   JCK-1
```

Mature Star

```
agcgucacgugauaacgcguccuuugcuuuuuauuuuuuauuugaaauucaaaaaaauaauaauaaagauauauuuauauaauuguggaugugaaagaagaacuacuaccacuacagagagauuuuuuuaugaggcugagaaaauuguuggcgugagagacuugguagaaaucuggguuuguuuggacacagaguuaggacuauucuucuuccgaguccgcccaugccaauaauuuccuccgucuuuuc
.................................................................................................................................................................................................................uuccgaguccgcccaugccaau..................   10   0   LCK-3
.................................................................................................................................................................................................................uuccgaguccgcccaugcAaau..................   1    1   JCK-3
.................................................................................................................................................................................................................uuccgaguccgcccaugccaCu..................   1    1   JCK-2
.................................................................................................................................................................................................................uuccgaguccgcccaugccaauU.................   24   1   JCK-2
.................................................................................................................................................................................................................uuccgaguccgcccaugccaauU.................   27   1   JCK-3
.................................................................................................................................................................................................................uuccgaguccgcccaugccaauU.................   16   1   JCK-1
.................................................................................................................................................................................................................uuccgaguccgcccaugccaauU.................   8    1   LT-2
.................................................................................................................................................................................................................uuccgaguccgcccaugccaauU.................   4    1   LCK-3
.................................................................................................................................................................................................................uuccgaguccgcccaugccaauU.................   17   1   LT-3
.................................................................................................................................................................................................................uuccgaguccgcccaugccaaua.................   1    0   LT-2
.................................................................................................................................................................................................................uuccgaguccgcccaugccaauU.................   23   1   LT-1
.................................................................................................................................................................................................................uuccgaguccgcccaugccaauUa................   3    1   JCK-3
.................................................................................................................................................................................................................uuccgaguccgcccaugccaauUa................   4    1   JCK-2
.................................................................................................................................................................................................................uuccgaguccgcccaugccaauUa................   2    1   JCK-1
.................................................................................................................................................................................................................uuccgaguccgcccaugccaauUa................   1    1   LT-3
.................................................................................................................................................................................................................uuccgaguccgcccaugccaauUau...............   1    1   LT-1
.................................................................................................................................................................................................................uuccgaguccgcccaugccaauaUu...............   1    1   LT-1
..................................................................................................................................................................................................................uccgaguccgcccaugcc.....................   1    0   LT-1
..................................................................................................................................................................................................................uccgaguccgcccaugcc.....................   1    0   JCK-3
..................................................................................................................................................................................................................uccgaguccgcccaugcca....................   1    0   LT-1
..................................................................................................................................................................................................................uccgaguccgcccaugcca....................   1    0   JCK-3
..................................................................................................................................................................................................................uccgaguccgcccaugccaa...................   2    0   LT-2
..................................................................................................................................................................................................................uccgaguccgcccaugccaau..................   2    0   JCK-2
..................................................................................................................................................................................................................uccgaguccgcccaugccaau..................   2    0   JCK-3
..................................................................................................................................................................................................................uccgaguccgcccaugccaau..................   1    0   LT-2
..................................................................................................................................................................................................................uccgaguccgcccaugccaaua.................   4    0   JCK-1
..................................................................................................................................................................................................................uccgaguccgcccaugccaaua.................   5    0   JCK-2
..................................................................................................................................................................................................................uccgaguccgcccaugccaaua.................   2    0   JCK-3
..................................................................................................................................................................................................................uccgaguccgcccaugccaauU.................   1    1   JCK-2
...................................................................................................................................................................................................................ccgaguccgcccaugccaaua.................   2    0   JCK-3
...................................................................................................................................................................................................................ccgaguccgcccaugccaaua.................   1    0   JCK-2
..................................................................................................................................................................................................................................ccaauaauuuccuccgucu....   1    0   LT-3
```
